# Supplementary material for: Image steganography without embedding by carrier secret information for secure communication in networks
Source: PLoS One. 2024 Sep 6;19(9):e0308265. doi: 10.1371/journal.pone.0308265 (PMC11379290; doi:10.1371/journal.pone.0308265)
Supplement: S1 File — (DOCX) [file pone.0308265.s001.docx]

In the article, we used three datasets: Bedroom, Churches, and FFHQ. Bedroom and Churches are from LSUN. In order to facilitate researchers in reproducing and researching our article, we have provided the references and download address of the dataset below. If you need any further information or assistance, please feel free to contact us at any time.

Paper:

Bedroom, Churches(LSUN): Lsun: Construction of a large-scale image dataset using deep learning.

FFHQ: A style-based generator architecture for generative adversarial.

Download address:

Bedroom, Churches(LSUN): https://github.com/fyu/lsun?tab=readme-ov-file

FFHQ: https://github.com/lulin60/FFHQdataset-paper
