# Supplementary material for: Image steganography without embedding by carrier secret information for secure communication in networks
Source: PLoS One. 2024 Sep 6;19(9):e0308265. doi: 10.1371/journal.pone.0308265 (PMC11379290; doi:10.1371/journal.pone.0308265)
Supplement: S2 File — (DOCX) [file pone.0308265.s002.docx]

We appreciate the emphasis on transparency and reproducibility in the research. We have ensured that all code generated by authors supporting the findings in our manuscript can be used without restrictions. Our code repository can be accessed through the following link: https://github.com/ywzhanggz/source-code. We believe that sharing our code in this way is in line with best practices and helps with reproduction and reuse. If you need any further information or assistance, please feel free to contact us anytime.
