# Supplementary material for: Image steganography without embedding by carrier secret information for secure communication in networks
Source: PLoS One. 2024 Sep 6;19(9):e0308265. doi: 10.1371/journal.pone.0308265 (PMC11379290; doi:10.1371/journal.pone.0308265)
Supplement: Attachment — Submitted filename: Response to Reviewers.docx [file pone.0308265.s003.docx]

RESPONSE LETTER

**Manuscript Number:** PONE-D-24-16179

**Title:** “Image steganography without embedding by carrier secret information for secure communication in networks”

**Authors:** Yangwen Zhang, Yuling Chen, Hui Dou, Chaoyue Tan, Yun Luo, Haiwei Sang

Dear editor and reviewers,

Thank you very much for your time involved in reviewing the manuscript. On behalf of all the contributing authors, I would like to express our sincere appreciations of your constructive comments concerning our article entitled “Image Steganography Without Embedding by Carrier Secret Information for Secure Communication in Networks” (Manuscript No.: PONE-D-24-16179). These comments are all valuable and helpful for improving our article. According to your comments, we have made extensive modifications to our manuscript to make our results convincing.

In addition, we promise to continuously revise our paper until all reviewers and the editor are satisfied. The following are the comments, our responses, and the detailed revisions that we have made. Finally, we would like to thank all of you very much for your constructive comments and positive support for this manuscript.

**Response to Reviewers:**

I. **Response to Review 1**

**Comment 1:** The abstract needs substential revision. Please write the abstact as: 2 lines with introducing the work, next 2 lines with presenting the peoblem and objectives, and then 2-3 lines discussing the methods and results.

**Response 1:** Thank you very much for the reviewers' suggestions. In response to your question, we have carefully revised the abstract section of the article. The first 2 lines introduced the work of image without embedding steganography. In the next 2 lines, we will introduce the existing problems of image without embedding steganography and the objectives of this article. In the last 3 lines, we discussed the method proposed in this article and its effectiveness. With the following modifications in the manuscript：

**(Abstract) (Page 1)**

Steganography, the use of algorithms to embed secret information in a carrier image, is widely used in the field of information transmission, but steganalysis tools built using traditional steganographic algorithms can easily identify them. Steganography without embedding (SWE) can effectively resist detection by steganography analysis tools by mapping noise onto secret information and generating secret images from secret noise. However, most SWE still have problems with the small capacity of steganographic data and the difficulty of extracting the data. Based on the above problems, this paper proposes image steganography without embedding carrier secret information. The objective of this approach is to enhance the capacity of secret information and the accuracy of secret information extraction for the purpose of improving the performance of security network communication. The proposed technique exploits the carrier characteristics to generate the carrier secret tensor, which improves the accuracy of information extraction while ensuring the accuracy of secret information extraction. Furthermore, the Wasserstein distance is employed as a constraint for the discriminator, and weight clipping is introduced to enhance the secret information capacity and extraction accuracy. Experimental results show that the proposed method can improve the data extraction accuracy by 10.03% at the capacity of 2304 bits, which verifies the effectiveness and universality of the method. The research presented here introduces a new intelligent information steganography secure communication model for secure communication in networks, which can improve the information capacity and extraction accuracy of image steganography without embedding.

**Comment 2:** The literature review needs to be strenghtened.

**Response 2:** Thank you very much for your suggestions. Based on your suggestion, we have made modifications and enhancements to the literature review section. It mainly enriches the content of the literature review and expresses the advantages and limitations of its work on the content of the literature review. In addition, we also discussed the advantages and limitations of existing work in related work to enhance the novelty and advantages of the proposed methods in the manuscript. The corresponding changes in the manuscript are as follows:

**(Lines 2 to 11) (Page 1)**

Information hiding techniques include steganography [1, 2] and watermarking [3, 4]. Digital watermarking is the process of embedding a watermark into a cover image by means of an algorithm or a deep neural network, which is used to protect the copyright of an image without the human eye being able to detect the watermark. Aditya [4] proposes a logistic map based fragile watermarking technique, which takes advantage of the sensitivity property of the logistic map to generate the watermark bits. Steganography is the process of hiding secret messages within a medium to create a steganographic carrier, which is then used to secure communication in networks [5], where steganography using images as the carrier medium is known as image steganography.

**(Lines 64 to 66 and Lines 84 to 104) (Page 3)**

The first strategy is to generate the corresponding hash sequences [17, 18] through a mapping association mechanism that involves the secret message and the set of available images.

The majority of existing SWE methods are afflicted by a number of shortcomings. These include the quality of steganographic images, which is often insufficient to achieve the desired effect of cover images; the capacity of steganographic information in steganographic images, which is often limited; and the accuracy of steganographic information extraction, which is frequently inadequate. Inspired by [22], we propose the carrier secret message method, which introduces a carrier component into the process of mapping secret message to secret noise. The properties of the carrier component enable the generation of carrier secret noise, which in turn enhances the capacity of the secret message to generate steganography images. Martin et al. [23] proposed that the Wasserstein distance enables the computation of the full set of all possible joint distributions. This allows for the estimation of the expected distance between samples to the distribution of the dataset, even when the data is unevenly distributed. Inspired by [24], we use the Wasserstein distance is integrated into the loss function of the discriminator. This enables us to assess the joint distribution between the cover image and the generated image, thereby enabling the generator to balance the quality of the steganographic image and the steganographic capacity of the secret message. Furthermore, inspired by [25], we believe that it is difficult for an unconstrained discriminator to balance the relationship between the quality of the generated image and the accuracy of secret message extraction. In order to address this issue, we use weight clipping, which enables the generated image to achieve a balance between the quality of the generated image and the accuracy of secret message extraction.

**(Lines 135 to 182) (Page 4),**

With the increasing awareness of network user security, secure network communication [27, 28] of message has received widespread attention. Image steganography has received widespread attention from scholars due to its characteristics. At the present time, two main types of image steganography [29]: traditional image steganography and deep learning [30, 31] based image steganography. Image steganography is able to hide secret message in cover images. It is a widely employed method in the fields of message security transmission [32–34], privacy protection [35], and copyright protection [36].

Ma et al. [37] proposed a hierarchical embedding RDHEI method that is capable of generating hierarchical labels from plaintext image distributions. This method categorizes hierarchical labels into three distinct categories: small, medium, and large. The labels are then used to categorize the types of plaintext images. Through predictive technology, hierarchical label maps are calculated before image steganographic embedding can increase the payload capacity while ensuring full reversibility of data. The secret information is precompressed and embedded into the carrier image. In comparison to previous spatial domain technologies, the RDHEI method of hierarchical embedding exhibits a higher load capacity. However, the method employs image steganography in the spatial domain, which will result in the loss of secret information in the steganography image if robustness attacks are performed on the steganography image.

The traditional image steganography of frequency domain transformation hides the secret data within the frequency domain of the cover image, which can significantly decrease the drop of secret data caused by robustness attacks. Giboulot Q et al [38] propose JPEG-based steganography and quantization table modification, by segmenting the carrier image into 8x8 pixel non-overlapping blocks and converting each non-overlapping into DCT coefficient by DCT respectively and designing an encryption algorithm for information encryption to become secret information. Conversion of spatial domain into frequency domain into 2D cosine wave. The secret information is then concealed within the DCT ratio, according to a pre-designed algorithm. However, the method generates steganography images that are susceptible to being identified by a steganography analysis tool when the method is subjected to a deep neural network steganography analysis tool that has been trained against it will leading to insecure transmission of secret information.

Mandal P C et al. [39] proposed an integer wavelet transform (IWT)-based steganography that uses the LSB method and coefficient value difference to utilize wavelet coefficients in approximate and diagonal subbands and in horizontal and vertical subbands, respectively. Since the perceptual range of the low-frequency coefficient is highly sensitive to the human eye, in this method, a small amount of secret information is embeded in the low-frequency coefficient to reduce the imperceptibility of the hidden image to the human eye after the image is hidden, and the threshold is set to ensure that the generated steganographic image has better visual quality. Nevertheless, the quality of the steganography image is guaranteed by the threshold setting method employed in the method, which results in the overall scheme being overly reliant on the expertise of the experts, thereby reducing its versatility.

3.	Luo L, Chen Z, Chen M, Zeng X, Xiong Z. Reversible image watermarking using interpolation technique. IEEE Transactions on information forensics and security. 2009;5(1):187–193.

4.	Sahu AK. A logistic map based blind and fragile watermarking for tamper detection and localization in images. Journal of Ambient Intelligence and Humanized Computing. 2022;13(8):3869–3881

17. Zhang X, Peng F, Long M. Robust coverless image steganography based on DCT and LDA topic classification. IEEE Transactions on Multimedia. 2018;20(12):3223–3238.

18. Zou L, Sun J, Gao M, Wan W, Gupta BB. A novel coverless information hiding method based on the average pixel value of the sub-images. Multimedia tools and applications. 2019;78:7965–7980.

22.	Ren W, Xu Y, Zhai L, Wang L, Jia J. Fast carrier selection of JPEG steganography appropriate for application. Tsinghua Science and Technology. 2020;25(5):614–624.

23.	Arjovsky M, Chintala S, Bottou L. Wasserstein GAN. 2017;.

24.	Gulrajani I, Ahmed F, Arjovsky M, Dumoulin V, Courville AC. Improved training of wasserstein gans. Advances in neural information processing systems. 2017;30.

35.	Sahu AK, Gutub A. Improving grayscale steganography to protect personal information disclosure within hotel services. Multimedia Tools and Applications. 2022;81(21):30663–30683.

36.	Sahu AK, Sahu M, Patro P, Sahu G, Nayak SR. Dual image-based reversible fragile watermarking scheme for tamper detection and localization. Pattern Analysis and Applications. 2023;26(2):571–590.

**Comment 3:** The research issue and objective of the work is missing.

**Response 3:** Many thanks to the reviewers for their suggestions, which have greatly enhanced our article.

Based on your feedback, we have added the research questions and objectives of this work in the introduction section. Our work mainly focuses on the research problem of low secret information capacity and poor extraction accuracy in the field of image steganography without embedding. Our work can improve the capacity and extraction accuracy of secret information while ensuring certain security conditions. Specific changes in the manuscript are listed below:

**(Lines 12 to 25) (Page 2)**

In the field of steganography without embedding, steganography without embedding is able to effectively resist the recognition of steganography analysis tools due to the fact that it does not directly employ the embedding of secret message into the cover image, thus achieving the purpose of securely transmitting information. The field of image steganography without embedding has attracted considerable interest among researchers due to its characteristics. However, the majority of studies on image steganography without embedding have been unable to effectively enhance the capacity and extraction accuracy of secret message while maintaining security. Previous methods have been to have limitations in terms of image distortion and the accuracy of message extraction, particularly when dealing with large capacities of steganographic message. This has led to a reduction in the usability of image steganography without embedding. This paper examines the potential of image steganography without embedding, with the objective of enhancing the capacity and extraction accuracy of secret message while maintaining a certain level of security.

**Comment 4:** The recent and related works should be discussed with its merits and issues: DOI: 10.4018/IJDCF.318666, https://doi.org/10.1007/s12652-021-03365-9, https://doi.org/10.3390/electronics12051222, Digital image steganography techniques in spatial domain: A study, https://doi.org/10.1016/j.jisa.2023.103541

**Response 4:** Thank you very much for the problems pointed out by the reviewers, which have greatly contributed to enhancing our article. Based on your feedback, carefully read the work of the above article and discuss its advantages and issues in our manuscript. In addition, we have read the follow-up work of the aforementioned paper, including DOI: <https://doi.org/10.1016/j.jksuci.2019.07.004>, https://doi.org/10.1007/s11042-022-13015-7, and https://doi.org/10.1007/s10044-022-01104-0, and also discussed the advantages and issues of their work in the manuscript. Specific changes in the manuscript are listed below:

**(Lines 2 to 11) (Page 1)**

Information hiding techniques include steganography [1, 2] and watermarking [3, 4]. Digital watermarking is the process of embedding a watermark into a cover image by means of an algorithm or a deep neural network, which is used to protect the copyright of an image without the human eye being able to detect the watermark. Aditya [4] proposes a logistic map based fragile watermarking technique, which takes advantage of the sensitivity property of the logistic map to generate the watermark bits. Steganography is the process of hiding secret messages within a medium to create a steganographic carrier, which is then used to secure communication in networks [5], where steganography using images as the carrier medium is known as image steganography.

**(Lines 26 to 45) (Page 2)**

Traditional image steganography is mainly based on algorithms for embedding of secret information into the carrier image, which is the main part of image steganography. The embedding method is divided into the spatial domain and frequency domain. Representatives of traditional steganography in the spatial domain are the LSB [6, 7], SUNIWARD [8]. Samar et al. [9] proposed a data hiding method that exploits the low embedding capacity and high variability properties of block-wise histogram shifting, thereby enhancing the robustness and embedding capacity of steganography. However, the spatial domain image steganography method will cause the pixel to change value in the steganographic image after the steganographic image is subjected to operations such as cropping, rotating, and scaling, and the steganographic information of the steganographic image will be lost. Traditional frequency domain image steganography hides the steganography in the frequency domain of the carrier image, which significantly reduces the secret information caused by image alteration. Representatives of traditional image steganography in frequency domain space include the discrete cosine transform (DCT) algorithm [10] and so on. Frequency domain steganography algorithms have better robustness compared to spatial domain steganography algorithms and can effectively resist robustness attacks. However, with the advent of steganalysis tools, as proposed by J. Hemalatha et al. [11] proposed the third order subtractive pixel adjacency matrix features with an ensemble classifier steganalysis method, which is able to effectively differentiate the distribution of steganographic images from clean images.

**(Lines 135 to 142) (Page 4)**

With the increasing awareness of network user security, secure network communication [27, 28] of message has received widespread attention. Image steganography has received widespread attention from scholars due to its characteristics. At the present time, two main types of image steganography [29]: traditional image steganography and deep learning [30, 31] based image steganography. Image steganography is able to hide secret message in cover images. It is a widely employed method in the fields of message security transmission [32–34], privacy protection [35], and copyright protection [36].

**(Lines 184 to 190) (Page 5)**

Goodfellow et al. [20] proposed a method for generating confrontation network GANs, which utilise a generator network and a discriminator network to combat the generation of images. The generator generates the image by inputting random noise, while the discriminator identifies the authenticity of the generated image. Both are trained against each other in order to improve the quality of the generated image. Furthermore, Vijay et al. [40] explored the current state of development of image steganography based on deep learning, which provides a viable direction for subsequent researchers.

3.	Luo L, Chen Z, Chen M, Zeng X, Xiong Z. Reversible image watermarking using interpolation technique. IEEE Transactions on information forensics and security. 2009;5(1):187–193.

4.	Sahu AK. A logistic map based blind and fragile watermarking for tamper detection and localization in images. Journal of Ambient Intelligence and Humanized Computing. 2022;13(8):3869–3881

7.	Sahu AK, Swain G. High fidelity based reversible data hiding using modified LSB matching and pixel difference. Journal of King Saud University-Computer and Information Sciences. 2022;34(4):1395–1409.

9.	Kamil Khudhair S, Sahu M, KR R, Sahu AK. Secure reversible data hiding using block-wise histogram shifting. Electronics. 2023;12(5):1222.

11.	Hemalatha J, Sekar M, Kumar C, Gutub A, Sahu AK. Towards improving the performance of blind image steganalyzer using third-order SPAM features and ensemble classifier. Journal of Information Security and Applications. 2023;76:103541.

35.	Sahu AK, Gutub A. Improving grayscale steganography to protect personal information disclosure within hotel services. Multimedia Tools and Applications. 2022;81(21):30663–30683.

36.	Sahu AK, Sahu M, Patro P, Sahu G, Nayak SR. Dual image-based reversible fragile watermarking scheme for tamper detection and localization. Pattern Analysis and Applications. 2023;26(2):571–590.

40.	Kumar V, Sharma S, Kumar C, Sahu AK. Latest trends in deep learning techniques for image steganography. International Journal of Digital Crime and Forensics (IJDCF). 2023;15(1):1–14.

**Comment 5:** Present the embedding and extraction process in an algorithmic way.

**Response 5:** Many thanks to the reviewers for pointing out the issues, which led to the enhancement of our article. Based on the shortcomings you pointed out, we have reorganized the content of the method in the embedding and extraction processes in the methods section, and added the embedding process of carrier secret noise (Algorithm 1) and the extraction process of carrier secret noise (Algorithm 2) to enhance the readability of the article. In addition, we also revised the content and formulas related to the generation and extraction of carrier secret noise in articles 3.1.1 and 3.1.2 to enhance the readability of the article. The corresponding changes in the manuscript are as follows:

**(Lines 273 to 278) (Page 8)**

| **Algorithm 1** | Carrier Noise Generation Algorithm |
| --- | --- |
| **Input:** | Secret message $M$ , Carrier noise $f (t_{m})$, Cover Image $I_{\mathrm{co}}$, Extractor $E_{s}$,  Structure generator $G_{\mathrm{stu}}$, Stego image generator $G$ |
| **Output:** | Stego Image $I_{\mathrm{st}}$ |
| 1: | The secret message $M$ is mapped to the secret tensor $Z$ according to Eq.(1) |
| 2: | The secret tensor $Z$ is generated according to Eq.(2) and carrier noise f (tm) to  generate the carrier secret tensor $Z'$ |
| 3: | Extractor $E_{s}$ obtains texture information $T_{1}$ and structural information $S_{1}$ from the cover image from $I_{\mathrm{co}}$ |
| 4: | The structural generator $G_{\mathrm{stu}}$ generates structural information $S_{2}$ through the secret tensor $Z'$ |
| 5: | Obtaining texture information $T_{2}$ through uniform distribution sampling |
| 6: | Input $G(T_{1},T_{2}, S_{1},S_{2})$ generates steganography images $I_{\mathrm{st}}$ |
| 7: | **return** $I_{st}$ |

The loading of carrier noise into the secret noise serves to restore the diversity of the secret noise, thereby enhancing the extraction accuracy of the secret message. The carrier noise generates the structural information $S_{2}$, which contains the noise $Z'$ through the generator $G_{\mathrm{stu}}$. The structural information, in conjunction with the texture information, generates the steganography image $I_{\mathrm{st}}$ through the image generator $G$. The process can be expressed as follows:

$$\begin{aligned} S_{2}=G_{\mathrm{stu}}\left( Z^{'} \right)\#\left( 4 \right) \end{aligned}$$

$$\begin{aligned} I_{st}=G\left( T_{1},T_{2},S_{1},S_{2} \right)\#\left( 5 \right) \end{aligned}$$

In the part of extracting secret message $M'$, the structural information extractor $E$ is responsible for the extraction of the $I_{st}$ generated by the generator $G$, which in turn generates the structural information $S'$ generated by the carrier noise. The structural information $S'$ is then extracted by the secret tensor extractor $E_{x}$ to obtain the carrier secret tensor $Z'’$, The extraction process of the carrier secret tensor is shown in Fig 4. The process can be expressed as:

$$\begin{aligned} S^{'}=E\left( I_{st} \right)\#\left( 6 \right) \end{aligned}$$

$$\begin{aligned} Z^{''}=E_{x}\left( S^{'} \right)\#\left( 7 \right) \end{aligned}$$

**(Lines 299 to 305) (Page 10)**

| **Algorithm 2** | Carrier Noise Extraction Algorithm |
| --- | --- |
| **Input:** | Stego image $I_{st}$, Carrier noise $f (t_{m})$, Structural information extractor $E$,  Extractor $E_{x}$ |
| **Output:** | Extraction secret message $M'$ |
| 1: | Extraction of structural information $S'$ in Ist by structural information extractor $E$ according to Eq.(6) |
| 2: | The extracted structural information $S'$ by the extractor $E_{x}$ to extract the carrier secret tensor $Z''$ |
| 3: | The $Z''$ is obtained by eliminating the $f (t_{m})$ according to Eq.(8) to obtain the extracted secret tensor $Z'''$ |
| 4: | The extracted secret tensor $Z'''$ is obtained by inverse mapping the extracted secret  meassge $M'$ according to Eq.(9) |
| 5: | **return** $M'$ |

The inverse carrier cancellation process is expressed as:

$$\begin{aligned} Z^{'''}=Z^{''}-f\left( t_{m} \right)\#\left( \mathbf{8} \right) \end{aligned}$$

The inverse mapping function process is expressed as:

$$\begin{aligned} M'=floor\left[ \left( Z^{'''}+1 \right)\times2\sigma-1 \right]\#\left( \mathbf{9} \right) \end{aligned}$$

The extraction of secret message through the secret tensor Z′′′ involves the removal of the carrier component and the utilisation of the asymmetric range extraction method for the remaining secret message tensor. This method has the capacity to enhance the accuracy of the extracted message, thereby increasing the efficiency and capacity of the transmission of secret message.

**Comment 6:** Provide numerical illustration.

**Response 6:** Many thanks to the reviewers for their suggestions. Based on your feedback, we have provided a detailed description of the image inputs X1, X2, and X3 of the extractor in the method, where the number of iterations is 80% of the total training times, depending on the variation in iteration times. In addition, we supplemented our selection of experimental values in weight cropping experiment 4.2. The revised parts of the corresponding manuscript are as follows:

**(Lines 379 to 381) (Page 12)**

The selection of hyperparameters for the number of iterations in the upper and lower sections of this reference is based on the numerical choice of hyperparameters for the number of iterations in IDEAS proposed by Liu et al. [42].

**(Lines 454 to 462) (Page 13)**

In selecting the size of the weight clipping, we set the hidden data volume with a conditional steganography capacity of 2304bits (N=3, σ=3) on the subset Bedroom in the LSUN dataset. Because the steganography capacity of 2304bits is currently medium level of steganography capacity in the field of SWE, it is able to more accurately reflect the scheme's ability to generate steganography image quality and secret message extraction. We then perturbed ∆=25 to perform weight clipping for range $C$ experiments. The weight clipping ranges selected for comparative experiments are (-0.1, 0.1), (-1.0, 1.0), (-2.0, 2.0), (-3.0, 3.0), (-4.0, 4.0), (-5.0, 5.0 ) and the unrestricted weight selection method for comparative experiments.

**Comment 7:** Validate the results with proper experimentation and sufficient performance measuring parameters.

**Response 7:** Many thanks to the reviewers for pointing out the issues, which led to the enhancement of our article. Based on the issues you pointed out in the experiment, we have supplemented the experimental content regarding the comparative experiment (Table 5) and the ablation experiment (Table 6 and 7). In the comparative experiment, we set hyperparameters ∆=50, ∆=25, and ∆=0 in Bedroom, Churches, and FFHQ datasets, and conducted a comparative experiment with a steganography capacity of 3072 bits. The superiority of our method over other methods has been verified through sufficient comparative experiments. In addition, in the ablation experiment, we validated the effectiveness and universality of all innovative points in our proposed method under different dataset conditions. The revised parts of the corresponding manuscript are as follows:

**(Lines 529 to 534) (Page 16)**

We compare WGAN-Steg, SAGAN-Steg, DCGAN-Steg, SSteGAN and IDEAS with our method under different steganographic capacities. The steganography capacity of the model includes 100bits, 200bits, 256bits (N=1, σ=1), 2304bits (N=3, σ=3), 3072bits (N=3, σ=4) respectively. In addition to that, we still set up the environment with different ∆ superparameters for implementation, which includes ∆=50, ∆=25, ∆=0.

Table 5. Compare the experimental results

|  | | Bedrooms | Churches | FFHQ | capacity |
| --- | --- | --- | --- | --- | --- |
| DCGAN-Steg | | 94.01% | 94.56% | 96.29% | 100bits |
| SAGAN-Steg | | 96.77% | 95.86% | 97.12% | 200bits |
| SSteGAN | | 98.41% | 97.53% | 97.23% | 100bits |
| WGAN-Steg | | 92.23% | 90.04% | 92.85% | 100bits |
| IDEAS | ∆=0 | 100% | 100% | 100% | 256bits |
|  | ∆=25 | 100% | 100% | 100% |  |
|  | ∆=50 | 99.54% | 99.55% | 99.49% |  |
|  | ∆=0 | 99.65% | 88.41% | 88.41% | 2304bits |
|  | ∆=25 | 86.15% | 99.48% | 83.80% |  |
|  | ∆=50 | 87.07% | 94.14% | 85.02% |  |
|  | ∆=0 | 75.78% | 95.05% | 87.11% | 3072bits |
|  | ∆=25 | 96.55% | 86.76% | 90.33% |  |
|  | ∆=50 | 87.50% | 88.12% | 85.42% |  |
| Ours | ∆=0 | 100% | 100% | 100% | 256bits |
|  | ∆=25 | 100% | 100% | 100% |  |
|  | ∆=50 | 100% | 100% | 100% |  |
|  | ∆=0 | 100% | 100% | 100% | 2304bits |
|  | ∆=25 | 100% | 100% | 100% |  |
|  | ∆=50 | 100% | 100% | 100% |  |
|  | ∆=0 | 100% | 100% | 100% | 3072bits |
|  | ∆=25 | 100% | 100% | 100% |  |
|  | ∆=50 | 100% | 100% | 100% |  |

**(Lines 545 to 564) (Page 17)**

We propose the innovation of Wasserstein distance as the loss function of the $D_{real}$, limit the weight range of the $D_{real}$, and load secret message on the carrier to improve the accuracy of message extraction. At the same time, we design an ablation experiment to prove our innovation points can achieve the expected effect when used alone, and the experimental results are shown in Table 6 and 7. It is verified that the carrier secret tensor, Weight clipping, and Wasserstein distance loss function can accurately improve the extraction rate of secret message. We performed ablation experiments with a steganography capacity of 2304bits (N = 3, σ = 3) and ∆ = 50 on the training set of Bedrooms, as shown in Table 6. In addition, we performed ablation experiments with a steganography capacity of 3072bits (N = 3, σ = 3) and ∆ = 25 on the training set of Churches, as shown in Table 7.

Table 7. Results of ablation experiments on the Churches dataset

| Carrier | Clamp | Wasserstein | ACC | Baseline(ACC) |
| --- | --- | --- | --- | --- |
| $\surd$ |  |  | 90.29% | 86.76% |
|  | $\surd$ |  | 91.48% |  |
|  |  | $\surd$ | 94.73% |  |
| $\surd$ | $\surd$ |  | 97.06% |  |
| $\surd$ |  | $\surd$ | 96.05% |  |
|  | $\surd$ | $\surd$ | 99.38% |  |
| $\surd$ | $\surd$ | $\surd$ | 100% |  |

Through Table 6 and 7, it is proved that the Wasserstein loss function proposed by us, the weight clipping limit $D_{real}$ discriminator and the carrier loaded secret information generation tensor can improve the accuracy of secret information extraction, and can be more effectively improved when combined with different methods extraction accuracy of secret information. The efficacy of the proposed carrier secret tensor, weight clipping, and Wasserstein distance loss function in enhancing the accuracy of secret message extraction can be demonstrated through the use of Table 6 and 7. Furthermore, the combination of these methods with different secret message extraction methods can result in enhanced effectiveness.

**Comment 8:** Mention the limitation and implications.

**Response 8:** We thank the reviewers for pointing out the problem. Based on your question, we have added a section on limitations and discussion in the article in section 6. In this section, we discuss the limitations and impacts of our method. Our method reduces the quality of steganographic images when increasing steganographic capacity, but it is insufficient when high capacity and high steganographic image quality are required. With the following modifications in the manuscript：

**(Lines 565 to 574) (Page 19)**

**5 Limitation and Discussion**

The proposed method is designed to enhance the steganography capacity and information extraction accuracy of unembedded images, thereby addressing the limitations of low steganography capacity and low extraction accuracy observed in unembedded images. Nevertheless, the scheme is still constrained by other issues. It is evident that the quality of steganography images in our proposed method still requires improvement, particularly in environments where high quality transmission is a necessity. In order to achieve high quality steganography images in low steganography capacity environments, it is necessary to reduce the size of the secret message. This allows for the generation of high quality steganography images for secure message transmission.

II. **Response to Review 2**

**Comment 1:** There are several grammatical errors and awkward phrasings throughout the manuscript. A thorough proofreading is necessary to improve readability. For example, "the covert information can increase the covert information capacity of steganography" could be rephrased for clarity.

**Response 1:** Thank you very much to the reviewer for pointing out the problem. Based on your feedback, we have thoroughly proofread the content of the article and improved its readability. In addition, based on the examples you provided in your feedback, we have made significant revisions to the abstract section of the article to improve its readability. The revised parts of the corresponding manuscript are as follows:

**(Abstract) (Page 1)**

Steganography, the use of algorithms to embed secret information in a carrier image, is widely used in the field of information transmission, but steganalysis tools built using traditional steganographic algorithms can easily identify them. Steganography without embedding (SWE) can effectively resist detection by steganography analysis tools by mapping noise onto secret information and generating secret images from secret noise. However, most SWE still have problems with the small capacity of steganographic data and the difficulty of extracting the data. Based on the above problems, this paper proposes image steganography without embedding carrier secret information. The objective of this approach is to enhance the capacity of secret information and the accuracy of secret information extraction for the purpose of improving the performance of security network communication. The proposed technique exploits the carrier characteristics to generate the carrier secret tensor, which improves the accuracy of information extraction while ensuring the accuracy of secret information extraction. Furthermore, the Wasserstein distance is employed as a constraint for the discriminator, and weight clipping is introduced to enhance the secret information capacity and extraction accuracy. Experimental results show that the proposed method can improve the data extraction accuracy by 10.03% at the capacity of 2304 bits, which verifies the effectiveness and universality of the method. The research presented here introduces a new intelligent information steganography secure communication model for secure communication in networks, which can improve the information capacity and extraction accuracy of image steganography without embedding.

**(Lines 2 to 25) (Page 1)**

Information hiding techniques include steganography [1, 2] and watermarking [3, 4]. Digital watermarking is the process of embedding a watermark into a cover image by means of an algorithm or a deep neural network, which is used to protect the copyright of an image without the human eye being able to detect the watermark. Aditya [4] proposes a logistic map based fragile watermarking technique, which takes advantage of the sensitivity property of the logistic map to generate the watermark bits. Steganography is the process of hiding secret messages within a medium to create a steganographic carrier, which is then used to secure communication in networks [5], where steganography using images as the carrier medium is known as image steganography.

In the field of steganography without embedding, steganography without embedding is able to effectively resist the recognition of steganography analysis tools due to the fact that it does not directly employ the embedding of secret message into the cover image, thus achieving the purpose of securely transmitting information. The field of image steganography without embedding has attracted considerable interest among researchers due to its characteristics. However, the majority of studies on image steganography without embedding have been unable to effectively enhance the capacity and extraction accuracy of secret message while maintaining security. Previous methods have been to have limitations in terms of image distortion and the accuracy of message extraction, particularly when dealing with large capacities of steganographic message. This has led to a reduction in the usability of image steganography without embedding. This paper examines the potential of image steganography without embedding, with the objective of enhancing the capacity and extraction accuracy of secret message while maintaining a certain level of security.

**(Lines 238 to 246) (Page 6)**

In this paper, we propose a framework for image steganography without embedding, in which secret message is hidden in generated images by carrier secret tensor. The carrier secret tensor can be effectively and accurately extracted from the generated image by an extractor. If the mapping interval in the input mapping noise of the network is too large, the accuracy of message extraction can be improved. Nevertheless, the singularity of noise can affect the diversity of generated images, which can result in a high degree of image overlap. Based on the above problems, the secret noise loaded on the carrier proposed by us can effectively solve the problem and enhance the accuracy of the message extraction process.

**(Lines 343 to 346) (Page 11)**

During the training process, if the weight value falls within the interval range of $(c_{\max}, c_{\min})$ , it is advisable to maintain the weight size p at its current value. However, if the weight size $p$ exceeds this range, it is necessary to assign a value to the weight and to limit the weight $p$ variety.

3.	Luo L, Chen Z, Chen M, Zeng X, Xiong Z. Reversible image watermarking using interpolation technique. IEEE Transactions on information forensics and security. 2009;5(1):187–193.

4.	Sahu AK. A logistic map based blind and fragile watermarking for tamper detection and localization in images. Journal of Ambient Intelligence and Humanized Computing. 2022;13(8):3869–3881

**Comment 2:** Ensure that all figures and tables are correctly labeled and referenced in the text. Some figures are referenced in a way that is not immediately clear.

**Response 2:** We thank the reviewers for their suggestions, which have greatly enhanced our article. Based on your feedback, we have thoroughly proofread the labeling and citation of images and tables in the article. We have modified the labeling and citation of images and tables in the article to ensure that each image and table is correctly labeled and cited in the article. Specific changes in the manuscript are listed below:

**(Lines 53 to 58) (Page 2)**

Nevertheless, the above is easily detected by steganalysis tools, which leads to the failure of transmission of the steganographic image, because the above methods hide the concealed messages by altering the pixels in the carrier image. Based on the above problems, a deep learning-based without embedding steganography method SWE [16] was developed. The steganography process of the SWE method is shown in Fig 1.

**(Lines 244 to 249) (Page6)**

Based on the above problems, the secret noise loaded on the carrier proposed by us can effectively solve the problem and enhance the accuracy of the message extraction process. Simultaneously, it can effectively avoid the situation that the generated image is too single due to the distribution of noise. The specific algorithms and flowcharts for the generation and extraction of carrier noise are provided in Algorithm 1 and Algorithm 2.

**(Lines 251 to 255) (Page 7)**

The current noise generation process in the SWE technique is to map the secret message M to be transmitted in the noise interval U through the mapping function, and the range of U is (-1, 1), to generate secret noise corresponding to the secret information. The secret message is mapped into σ-bits noise distribution segments for the mapping function. The overall process is shown in Fig 3.

**(Lines 285 to 289) (Page9)**

In the part of extracting secret message $M,$the structural information extractor $E$ is responsible for the extraction of the $I_{st}$ generated by the generator $G$, which in turn generates the structural information $S'$ generated by the carrier noise. The structural information $S'$ is then extracted by the secret tensor extractor $E_{x}$ to obtain the carrier secret tensor $Z'$. The extraction process of the carrier secret tensor is shown in Fig 4.

**(Lines 484 to 490) (Page15)**

In all methods of image steganography, it is necessary to ensure that the human eye cannot distinguish between the cover image and stego image during transmission. Consequently, the quality of the image generated by the method described in this paper must meet this indistinguishability requirement. This subsection presents verification of the quality of steganography images generated by our method. The FID index is the most widely used evaluation index for image generation. It is employed to evaluate the image quality of our proposed method. The experimental results are shown in Table 2.

**Comment 3:** The related work section is comprehensive but could benefit from a more critical analysis of the limitations of existing methods. This would better highlight the novelty and advantages of the proposed approach. Besides, the title of Section 2.2 does not align with the content discussed. It is suggested to refine the title to better reflect the scope of the section, e.g., Image Steganography Based on Cover Generation.

**Response 3:** Many thanks to the reviewers for their suggestions. Firstly, thank you for acknowledging the comprehensiveness of the relevant work sections in the manuscript. Based on your feedback, we have added the advantages and limitations of the existing methods mentioned in the relevant work section to enhance the novelty and advantages of our proposed method. In addition, based on your question about the lack of detailed titles in section 2.2 of the manuscript, we have re read and revised some of the chapter content, changing the title of section 2.2 to Image Steganography Based on Cover Generation. The corresponding changes in the manuscript are as follows:

**(Lines 147 to 220) (Page 5)**

Ma et al. [37] proposed a hierarchical embedding RDHEI method that is capable of generating hierarchical labels from plaintext image distributions. This method categorizes hierarchical labels into three distinct categories: small, medium, and large. The labels are then used to categorize the types of plaintext images. Through predictive technology, hierarchical label maps are calculated before image steganographic embedding can increase the payload capacity while ensuring full reversibility of data. The secret information is precompressed and embedded into the carrier image. In comparison to previous spatial domain technologies, the RDHEI method of hierarchical embedding exhibits a higher load capacity. However, the method employs image steganography in the spatial domain, which will result in the loss of secret information in the steganography image if robustness attacks are performed on the steganography image.

The traditional image steganography of frequency domain transformation hides the secret data within the frequency domain of the cover image, which can significantly decrease the drop of secret data caused by robustness attacks. Giboulot Q et al [38] propose JPEG-based steganography and quantization table modification, by segmenting the carrier image into 8x8 pixel non-overlapping blocks and converting each non-overlapping into DCT coefficient by DCT respectively and designing an encryption algorithm for information encryption to become secret information. Conversion of spatial domain into frequency domain into 2D cosine wave. The secret information is then concealed within the DCT ratio, according to a pre-designed algorithm. However, the method generates steganography images that are susceptible to being identified by a steganography analysis tool when the method is subjected to a deep neural network steganography analysis tool that has been trained against it will leading to insecure transmission of secret information.

Nevertheless, the quality of the steganography image is guaranteed by the threshold setting method employed in the method, which results in the overall scheme being overly reliant on the expertise of the experts, thereby reducing its versatility.

**2.2 Image Steganography Based on Generation Network**

Goodfellow et al. [20] proposed a method for generating confrontation network GANs, which utilise a generator network and a discriminator network to combat the generation of images. The generator generates the image by inputting random noise, while the discriminator identifies the authenticity of the generated image. Both are trained against each other in order to improve the quality of the generated image. Furthermore, Vijay et al. [40] explored the current state of development of image steganography based on deep learning, which provides a viable direction for subsequent researchers.

Yu et al. [41] proposed that the combination of GAN and image steganography in SGAN verifies the advantages of GAN over traditional image steganography and further improves the embedding ability, undetectability and robustness of steganographic images. SGAN generate images through generators, utilising secret information as input to generators in generative adversarial networks. The discriminator enhances the image produced and the ability of the discriminator to discriminate the input produced image and the original image. The extractor is introduced to extract and recover the secret information in the produced image. However, this scheme only explores the application of GAN in image steganography, which requires specific information data hiding is more challenging and the network model needs to be more carefully tuned to adapt to the secret information capacity of steganography and the quality of the generated images.

Arjovsky et al. [25] proposed the use of Wasserstein distances as a loss function in GANs, with measure the distance between the generated image and the cover image distribution. In contrast to the KL divergence and JS divergence, the Wasserstein distance can still reflect the distance relationship between two regions in the absence of overlapping regions or rarely overlap. The scheme explores the application of Wasserstein distance in GANs network and achieves promising results, but the small capacity of secret information of steganographic images generated by the scheme may lead to incomplete transmission of secret information under high-capacity secret information steganography conditions.

Liu et al. [42] proposed IDEAS, which extracts structural information and texture information from images through the decoupling of the image components. This method uses different structural information and texture information as the input of the training generator to generate steganographic images. Extensive experimentation has proven that conducted to demonstrate that the quality of secret images generated by decoupling information, the accuracy of extracted information and the quality of generated images are significantly enhanced in comparison to previous methods of SWE. Nevertheless, the scheme remains inadequate in its treatment of the relationship between the quality of steganography images and the accuracy of secret information extraction at higher capacity.

40.	Kumar V, Sharma S, Kumar C, Sahu AK. Latest trends in deep learning techniques for image steganography. International Journal of Digital Crime and Forensics (IJDCF). 2023;15(1):1–14.

**Comment 4:** The introduction should provide a clearer motivation. Why is the proposed method important, and what specific gap does it fill in the current literature?

**Response 4:** Many thanks to the reviewers for their suggestions. Based on your feedback, we have added the motivation behind our proposed improvement method in the introduction section to enhance the readability of the article and the importance of the proposed solution. With the following modifications in the manuscript：

**(Lines 84 to 104) (Page 3)**

The majority of existing SWE methods are afflicted by a number of shortcomings. These include the quality of steganographic images, which is often insufficient to achieve the desired effect of cover images; the capacity of steganographic information in steganographic images, which is often limited; and the accuracy of steganographic information extraction, which is frequently inadequate. Inspired by [22], we propose the carrier secret message method, which introduces a carrier component into the process of mapping secret message to secret noise. The properties of the carrier component enable the generation of carrier secret noise, which in turn enhances the capacity of the secret message to generate steganography images. Martin et al. [23] proposed that the Wasserstein distance enables the computation of the full set of all possible joint distributions. This allows for the estimation of the expected distance between samples to the distribution of the dataset, even when the data is unevenly distributed. Inspired by [24], we use the Wasserstein distance is integrated into the loss function of the discriminator. This enables us to assess the joint distribution between the cover image and the generated image, thereby enabling the generator to balance the quality of the steganographic image and the steganographic capacity of the secret message. Furthermore, inspired by [25], we believe that it is difficult for an unconstrained discriminator to balance the relationship between the quality of the generated image and the accuracy of secret message extraction. In order to address this issue, we use weight clipping, which enables the generated image to achieve a balance between the quality of the generated image and the accuracy of secret message extraction.

22.	Ren W, Xu Y, Zhai L, Wang L, Jia J. Fast carrier selection of JPEG steganography appropriate for application. Tsinghua Science and Technology. 2020;25(5):614–624.

23.	Arjovsky M, Chintala S, Bottou L. Wasserstein GAN. 2017;.

24.	Gulrajani I, Ahmed F, Arjovsky M, Dumoulin V, Courville AC. Improved training of wasserstein gans. Advances in neural information processing systems. 2017;30.

**Comment 5:** The terminologies should be standardized, e.g., "picture" should be consistently referred to as "image."

**Response 5:** Many thanks to the reviewers for pointing out the problem. Based on your feedback, we have thoroughly proofread the standardization issues of terminology in the manuscript. Change "picture" to "image" in the manuscript. In addition, we have proofread the manuscript regarding the secret message based on your feedback and revised the term "secret information" to "secret message" in the manuscript. The corresponding changes in the manuscript are as follows:

**(Lines 108 to 111) (Page 4)**

The enhanced discriminator for the training effect of the generator, the generator can better produce the steganography image by the steganography image by the secret message noise tensor mapped by the steganography information.

**(Lines 348 to 352) (Page 11)**

The Wasserstein distance structure uses the Wasserstein distance to measure the relationship between the cover image and the generated image. We calculate the smallest Wasserstein distance, which is as close as possible to the cover image distribution relationship. The cover image and the generated image are distinguished by the discriminator $D_{real}.$

**(Lines 484 to 488) (Page 15)**

In all methods of image steganography, it is necessary to ensure that the human eye cannot distinguish between the cover image and stego image during transmission. Consequently, the quality of the image generated by the method described in this paper must meet this indistinguishability requirement. This subsection presents verification of the quality of steganography images generated by our method.

**(Lines 502 to 509) (Page 15)**

Table 3 shows that the steganographic images generated by our method and the images generated by other benchmark methods are all around 0.5 for steganalysis tools, which means that for steganalysis tools, only cover images and generated images can be randomly judged. The authenticity of the image cannot be effectively distinguished. The results demonstrate that our method is resilient to detection by steganalysis tools and that it is challenging for such tools to discern whether the image generated by the SWE method is genuine. The transfer of secret message can be discovered by steganalysis tools, thus providing a reliable method for the transfer of secret message.
